# Frontal sinus adenocarcinoma

**DOI:** 10.1590/S1516-31802000000400009

**Published:** 2000-07-07

**Authors:** Márcio Abrahão, Ana Paula Vieira Gonçalves, Roberto Yamashita, Rogério Aparecido Dedivitis, Rodrigo Oliveira Santos, Luiz Augusto Nascimento, Marcelo Luis Mudo, Fernando Antonio Patriani Ferraz, Onivaldo Cervantes

**Keywords:** Frontal sinus adenocarcinoma, Head and neck neoplasm, Paranasal sinus cancer, Adenocarcinoma de seio frontal, Câncer de seios paranasais, Neoplasia de cabeça e pescoço

## Abstract

**CONTEXT:**

Paranasal sinus cancer is considered rare, with an incidence of less than 1 per 100,000 per year, with the frontal sinus being the primary site in only 0.3%. We report a case of adenocarcinoma arising in the frontal sinus.

**DESIGN:**

Case report.

**CASE REPORT:**

A 59-year-old woman, secretary, came in February 1998 with a 4-month history of low intensity frontal headache. She denied contact with wood dust. On examination a non-tender swelling was noted over her right forehead next to the medial aspect of the right orbit. CT scan showed a soft-tissue mass involving frontal sinus with intracranial invasion through the posterior wall. The anterior ethmoid sinus and the medial aspect of the right orbit were also involved. MRI demonstrated dural thickening in communication with the frontal mass. She underwent an en-bloc tumor resection by craniotomy including orbital clearance. Histology revealed an adenocarcinoma. After surgery she had tumor recurrence, and chemotherapy and radiotherapy were started resulting in partial improvement.

## RESUMO

**CONTEXTO:**

O câncer dos seios paranasais é considerado raro com uma incidência menor que 1 por 100.000 por ano. O seio frontal é o sítio primário em apenas 0,3% dos casos. A seguir relatamos um caso de adenocarcinoma do seio frontal.

**TIPO DE ESTUDO:**

Relato de caso.

**RELATO DO CASO:**

Paciente do sexo feminino, 59 anos, secretária, com queixa de quatro meses de cefaléia frontal de leve intensidade (fevereiro de 1998). Negava contato com serragem. Ao exame notava-se um abaulamento frontal à direita, adjacente a borda medial da órbita direita, de característica endurecida. A tomografia computadorizada mostrava um tumor com densidade de partes moles preenchendo o seio frontal com invasão intracraniana através da parede posterior. O seio etmoidal anterior e a borda medial da órbita direita também estava envolvida. A ressonância nuclear magnética demonstrava espessamento dural em comunicação com o tumor. A paciente foi submetida a exérese tumoral em bloco através de craniotomia bicoronal incluindo exenteração orbitária à direita. O exame histopatológico revelou adenocarcinoma. Após a cirurgia a paciente apresentou recidiva tumoral com resposta parcial a radioterapia e quimioterapia.

## INTRODUCTION

Cancer of the nasal cavity and paranasal sinuses is relatively rare with an incidence of less than 1 per 100,000 persons per year,^1^ and represents only 3 % of all head and neck malignancies.^2^^,^^3^ Among paranasal sinuses, the frontal sinus is one of the least affected (together with the sphenoid sinus) with an incidence of about 0.3 %.^2^ The symptoms are vague, simulating inflammatory diseases, which is why these tumors are often diagnosed at an advanced stage. This, in association with the complex anatomy of the region, turns such tumors into a therapeutic challenge.

The histologically predominant type, both in the nasal cavity and in paranasal sinuses, is squamous cell carcinoma (SCC), present in 43%-64% of the cases,^4,5^ with adenocarcinoma (AC) appearing as the second more frequent type, in 10 to 20 %.^2,4-6^ Nevertheless, in the frontal sinus, squamous cell carcinoma is far more frequent than adenocarcinoma with a ratio of about 20:1.^7^ Malignant tumors of respiratory tract are strongly associated with exogenous factors, especially from industrial processes such as production of chromium, nickel, mustard gas and polycyclic hydrocarbons, and with aflatoxins, which are associated with squamous cell carcinoma. The link between wood dust and adenocarcinoma of ethmoid sinuses is well known.^8^ We report here a case of frontal sinus adenocarcinoma where no occupational factor was found.

## CASE REPORT

A 59-year-old woman, a secretary, came in February 1998 with a 4-month complaint of low intensity frontal headache. At first it was attributed to sinusitis but antibiotics offered no relief. She denied tobacco abuse or contact with chemicals or wood dust. On examination a non-tender swelling was noted over her right forehead next to the medial aspect of the right orbit. Extraocular movements and rhinoscopy were normal.

**Figures 1:**
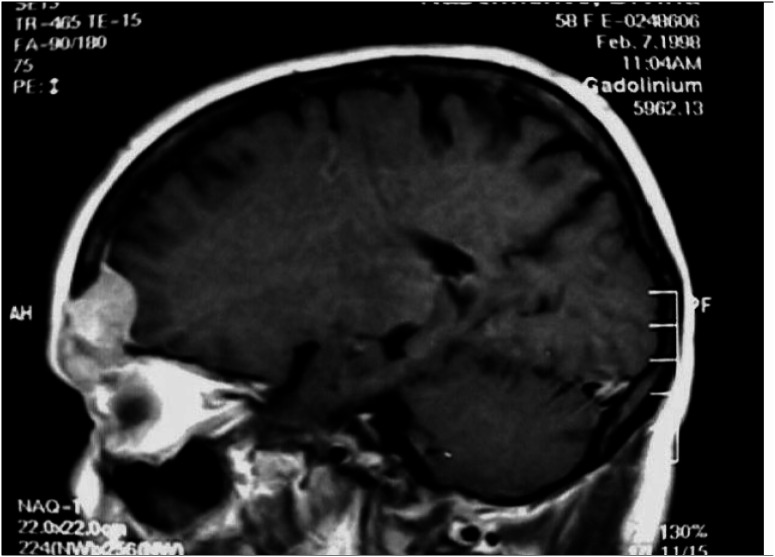
MRI demonstrated dural thickening in communication with the frontal mass.

**Figures 2:**
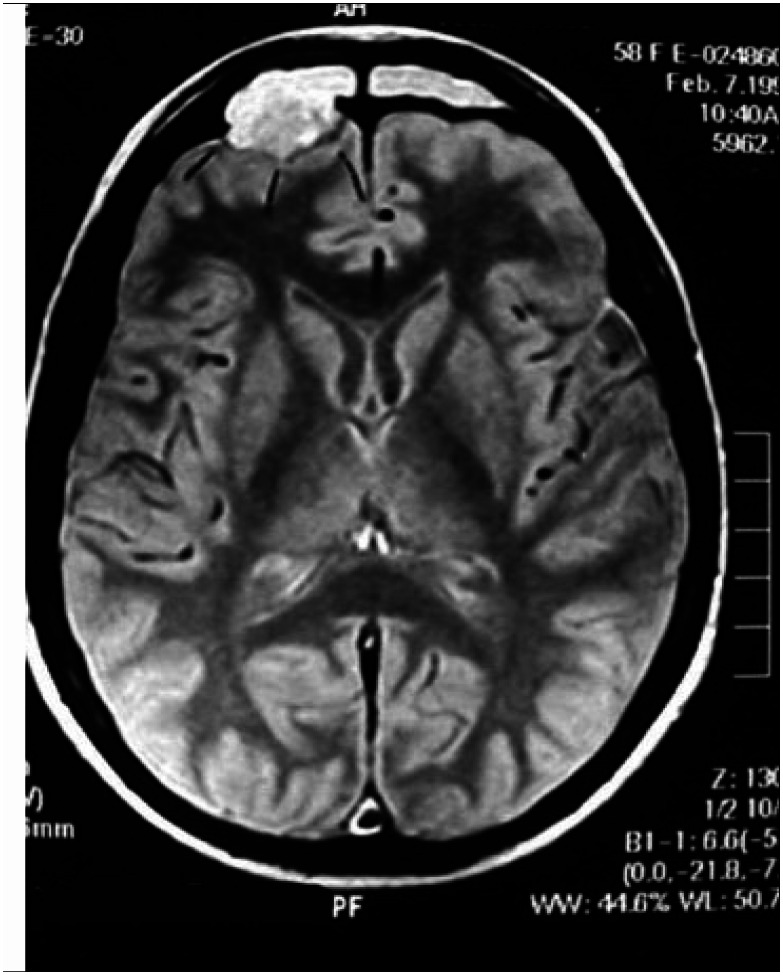
MRI demonstrated dural thickening in communication with the frontal mass.

She underwent a bicoronal craniotomy followed by shield-shaped frontal osteotomy. The frontal sinus was completely filled by tumor with extension to the anterior ethmoid cells, right lamina papyracea and dura-mater next to the posterior wall. An en-bloc resection was done including right orbital clearance. The anatomical defect was repaired with acrylic plate and galea. Histology revealed adenocarcinoma. Post-operatively she developed wound infection and the acrylic plate had to be removed, which postponed the initiation of adjuvant therapy. 45 days after surgery she developed recurrence above her left orbit. Chemotherapy with 5 fluorouracil and Taxol and radiotherapy was started which led to the disappearance of the tumor. Ten months after surgery she was still alive but had recurrence in the nasal cavity.

## DISCUSSION

In contrast to ethmoid sinuses, where adenocarcinoma is predominant, and in spite of the anatomical proximity, adenocarcinoma arising in the frontal sinus is an exception. Out of 772 cases of nasal sinus tumors, Lewis & Castro (1972) found 6 cases of cancer arising in the frontal sinus, all of which were squamous cell carcinoma.

Paranasal sinus carcinomas have a strong occupational association. In 1992, Nuñez et al. found a risk of developing paranasal adenocarcinoma 896 times higher in a population exposed to wood dust than in the general population. This probably explains why its male predominance is 1.7 times higher than among females.^2,4,6,9^ Our patient denied exposure to any kind of exogenous factors from industry. The peak incidence is when close to the sixth decade.^4,10^

The case reported here is a typical example of paranasal cancer, with a silent onset simulating a benign disease. The advanced stage of the tumors at presentation and the complex anatomy of the region make it difficult to perform resection with adequate margins.

**Figure 3:**
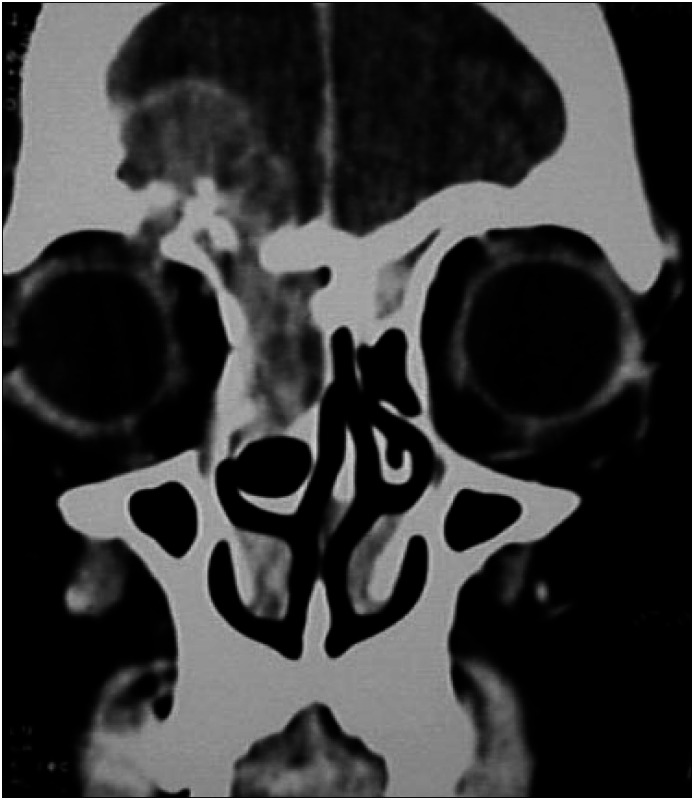
CT scan showed a soft-tissue mass involving the frontal sinus with intracranial invasion through the posterior wall. Anterior ethmoid air cells and the medial aspect of the right orbit were also involved.

There are no controlled trials to give assurance about how radiotherapy or chemotherapy affects survival, but these treatments are helpful in local control. The overall response to chemotherapy is about 82% and regimens based on cisplatin seem to be more effective, especially when associated to 5-fluorouracil and bleomycin.^11^

It is difficult to evaluate the prognostic due to rareness of nasal sinus tumors and diversity of treatment and histologic types. The overall survival over 5 years ranges from 31 to 46%.^4,5^ Apparently, adenocarcinoma has a better survival over 5 years (65%) than squamous cell carcinoma (35%),^12^ The case we report here had two predictors of poor outcome: intracranial involvement^13^ and orbital extension.

Patients submitted to craniofacial resection without orbital involvement have a 5-year survival of 69% compared with 29% for those with involvement, including periosteum alone.^13^ Our patient underwent orbital clearance, due to gross orbital extension, but preoperative radiation and frozen sections of periorbital involvement can in some instances be useful in preserving the eye without compromising oncological safety.^14^
